# Ultrasound Diagnosis and Guided Intervention of Musculoskeletal/Neuromuscular Pathology 2022

**DOI:** 10.3390/diagnostics13111945

**Published:** 2023-06-02

**Authors:** Ke-Vin Chang

**Affiliations:** 1 Department of Physical Medicine and Rehabilitation, National Taiwan University Hospital, Bei-Hu Branch, Taipei 10845, Taiwan; kvchang011@gmail.com; 2 Department of Physical Medicine and Rehabilitation, National Taiwan University College of Medicine, Taipei 10048, Taiwan; 3 Center for Regional Anesthesia and Pain Medicine, Wang-Fang Hospital, Taipei Medical University, Taipei 11600, Taiwan

The field of musculoskeletal medicine has been revolutionized by the introduction of ultrasound imaging. With its exceptional resolution, ultrasound facilitates the identification of soft tissue lesions, including tendinopathy, ligament sprains, and muscle tears [1]. Its portability enables real-time assessment of pain sources in clinics and at the patient’s bedside, making it highly accessible compared to magnetic resonance imaging. As a result, there is a growing body of research exploring the utility of ultrasound in musculoskeletal medicine [2].

Advancements in transducer frequency and imaging processing techniques have further enhanced the capabilities of ultrasound. Peripheral nerves, from surrounding connective tissues to inner nerve fascicles, can now be visualized with remarkable clarity [3]. Ultrasound has gained recognition for its diagnostic performance in detecting peripheral nerve entrapment [4]. To consolidate research efforts in this area, we organized a Special Issue titled “Ultrasound Diagnosis and Guided Intervention of Musculoskeletal/Neuromuscular Pathology 2022,” which featured 15 articles.

Six original articles utilized ultrasound for the quantification and qualification of muscles. Mateos-Angulo et al. [5] discovered significant associations between muscle thickness, echo intensity, and cognitive and physical dimensions in older adults, indicating that echo intensity adjusted by handgrip strength could indirectly indicate functional capacity. Ptaszkowski et al. [6] employed sonoelastography to identify differences in elastographic assessment of the pelvic floor between rest and contraction in patients with stress urinary incontinence. Sánchez Romero et al. [7] demonstrated the test–retest reliability of ultrasound thickness measurements and muscle contraction ratio, establishing ultrasound imaging as a highly reliable method for assessing transversus abdominis and lumbar multifidus thickness in dynamic positions. Additionally, Mateos-Angulo et al. [8] found significant differences in muscle thickness and echo intensity between post-polio patients and healthy controls, specifically in the rectus femoris muscle. Sicilia-Gomez et al. [9] observed excellent reliability in diaphragm thickness measurements during tidal volume and expiration at forced volume in subjects with or without low back pain. Lastly, Ortega-Santamaría et al. [10] utilized M-mode ultrasonography to evaluate various aspects of the infraspinatus muscle, measuring muscle thickness at rest and during contraction, as well as the velocity of muscle activation and relaxation. Muscle thickness measurements demonstrated good reliability, while activation and relaxation velocity showed moderate reliability.

In addition to muscle research, Lee et al. [11] conducted a study on adhesive capsulitis patients, noting significant decreases in the thickness of the axillary recess over a 6-month follow-up period. The thickness of the rotator interval showed a significant decrease only between the baseline and 1-month evaluation. Yau et al. [12] examined patients with carpal tunnel syndrome and found notable improvements in ultrasound parameters, global symptom score, and distal motor latency in the oral steroid group compared to the nicergoline group. Ultrasound changes were observable as early as 2 weeks following oral steroid treatment, indicating the potential of ultrasound as a non-invasive and effective tool for evaluating treatment effects for carpal tunnel syndrome.

The Special Issue also included five reviews. Chen et al. [13] provided detailed information on the anatomical structures of the forefoot, highlighting common pathologies affecting it. Lam et al. [14] conducted a systematic review and meta-analysis to assess the effectiveness of different regimens for ultrasound-guided intervention in carpal tunnel syndrome. Wu et al. [15] presented a systematic protocol for examining facial neurovascular structures and discussed their relevance to aesthetic injections. Wu et al. [16] further explored the anatomy of ischiofemoral spaces, utilizing cadaveric models and magnetic resonance imaging, and discussed the application of ultrasound in diagnosing and guiding injections for ischiofemoral impingement syndrome. Lastly, Wang et al. [17] provided an in-depth analysis of the sonoanatomy of the carpal bones and presented a systematic approach for navigating wrist ligaments, both extrinsic and intrinsic.

Furthermore, the Special Issue featured two case reports. Mezian et al. [18] described a unique case where ultrasound imaging played a crucial role in diagnosing and surgically managing a nonanatomic repair of ankle extensor tendons after a penetrating injury. Wu et al. [19] reported a case of a child with a lateral patellar retinaculum defect in pediatric knees, diagnosed through dynamic ultrasound. This case underscored the usefulness of ultrasound examination in pediatric knee problems and its ability to capture the exact “pathological moment” through dynamic scanning.

In conclusion, this Special Issue highlights the significant development of ultrasound imaging in musculoskeletal medicine. Both static and dynamic [20] ultrasound offer valuable tools for evaluating and diagnosing musculoskeletal and neuromuscular pathologies, benefiting clinicians and patients alike.

## Data Availability

Data are contained within the main text of the manuscript.
